# Tests of hypotheses for group formation in the subtropical leaf‐dwelling bat, *Kerivoula furva*

**DOI:** 10.1002/ece3.7524

**Published:** 2021-04-03

**Authors:** Chia‐Wei Hsu, Mei‐Ting Kao, Cheng‐Han Chou, Hsi‐Chi Cheng, Jian‐Nan Liu

**Affiliations:** ^1^ Department of Forestry and Natural Resources National Chiayi University Chiayi City Taiwan; ^2^ Endemic Species Research Institute Council of Agriculture Jiji Township Taiwan

**Keywords:** active aggregation, association index, dark woolly bat, social groups, vespertilionids

## Abstract

Investigating factors that promote group living in animals can help us to understand the evolution of sociality. The dark woolly bat, *Kerivoula furva*, forms small groups and uses furled leaves of banana (*Musa formosana*) as day roosts in subtropical Taiwan. In this study, we reported on the roosting ecology and social organization of *K. furva*. We examined whether ecological constraints, demographic traits, and physiological demands contributed to its sociality. From July 2014 to May 2016, we investigated the daily roost occupation rate, group size, and composition of each roost, and we calculated association indices in pairs. The results showed *K. furva* lived in groups throughout the year, and the average daily roost occupation rate was approximately 6.7% of all furled leaves that were suitable for roosting. The size of roosting groups of adults in each roost varied between 1 and 13; group size was independent of air temperature during both reproductive and nonreproductive seasons. The vast majority of roosting groups was composed of females and their young, and males frequently roosted solitarily or in a bachelor group. Forty adult bats were captured ≥4 times during the study period. The association indices in pairs of these 40 bats ranged between 0 and 0.83 with an average of 0.05 ± 0.14 (*n* = 780). The average association index of female–female pairs was significantly higher than that of female–male pairs and male–male pairs. Based on the association indices, the 40 bats were divided into seven social groups with social group sizes that varied between 2 and 10. Despite changing day roosts frequently, the relatively stable social bonds were maintained year‐round. Our results that groups of *K. furva* were formed by active aggregation of multiple generation members supported the demographic traits hypothesis.

## Graphical abstract

we examined whether ecological constraints, demographic traits, and physiological demands contributed to the group formation in *Kerivoula furva*, a little known species that use banana furled leaves as day roosts in subtropical regions. We found that *K. furva* maintained a relatively stable social groups year‐round, despite they switched roosts frequently. Our results that social groups of *K. furva* were formed by active aggregation of multiple generation members supported the demographic traits hypothesis for group formation in this species.

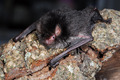

## 1 INTRODUCTION

Many mammals, such as primates, cetaceans, and elephants, live in groups and exhibit complex social behaviors (e.g., Kappeler & van Schaik, 2002; Whitehead, 2003; Archie et al., 2006). Group living facilitates social interactions and then drives sociality when group members increase individual inclusive fitness through social interactions such as cooperation, mutual thermoregulation, and anti‐predation (Alexander, 1974; Hamilton, 1964). Social organization, which is defined as size, sexual composition, and spatiotemporal cohesion of society, is an important aspect of society (Kappeler & van Schaik, 2002). Quantifying the social organization could provide valuable insight into the evolution of sociality (Hinde, 1976; Kappeler & van Schaik, 2002).

The Chiroptera represents the second most species‐rich order of mammals, with near 1,400 extant species (Burgin et al., 2018). Bats are distributed widely in most terrestrial habitats and use a wide variety of roost types from permanent to extremely ephemeral. The majority of bat species live in groups that range from a few individuals to millions (Kunz & Lumsden, 2003). The stability of groups in bats differs according to environmental conditions and roost characteristics (Sagot, 2016). For example, temperate bats generally exhibit mixed‐sex aggregations during hibernation and the mating season but usually demonstrate sexual segregation at parturition, in which reproductive females and pups form maternity colonies and males live solitarily or form bachelor groups (Bradbury, 1977; McCracken & Wilkinson, 2000; Sagot, 2016). In contrast, group stability was more flexible in tropical bats; some tropical species exhibited seasonally unstable groups, whereas some others, such as Spix's disk‐winged bats (*Thyroptera tricolor*) and greater sac‐winged bats (*Saccopteryx bilineata*), maintained stable social groups throughout the year (Kerth, 2008; McCracken & Wilkinson, 2000; Sagot, 2016).

Bats have been shown to exhibit complex and diverse social behaviors and mating systems (Kerth, 2008; McCracken & Wilkinson, 2000; Wilkinson et al., 2019). Due to their cryptic lifestyle, however, investigation of social interactions among free‐ranging bats is often challenging. Consequently, social organization and group stability have been quantified for only a small number of bat species and most studies have focused on vespertilionids from temperate regions (Kerth, 2008; Patriquin & Ratcliffe, 2016; Wilkinson et al., 2019). Although most tropical bats are social and exhibit extensive variation in group stability (Kerth, 2008; McCracken & Wilkinson, 2000), detailed studies on sociality have been reported in only a few species (Flores et al., 2020; Patriquin & Ratcliffe, 2016; Wilkinson et al., 2019). Little attention has been paid to the sociality study of subtropical bats, particularly in the Oriental Region.

Investigating the factors that promote group living of animals is fundamental to understanding the evolution of sociality. Kerth (2008) identified three factors that might contribute to the evolution of sociality in bats: (a) ecological constraints: Bats are forced to aggregate because suitable roosts were limited (Kunz & Lumsden, 2003), (b) demographic traits: The relatively long longevity and natal philopatry of bats result in multigenerational social groups (Kerth, 2008), (c) physiological demands: Bats gain energetic benefits from social thermoregulation (Pretzlaff et al., 2010; Russo et al., 2017; Willis & Brigham, 2007). Individual thermal benefit from group living might vary with sex, reproductive condition, and group size. In many species, reproductive female bats maintain normothermia to maximize offspring development (e.g., Pretzlaff et al., 2010; Willis & Brigham, 2007); thus, reproductive females could profit energetically from mutual warming by clustering (e.g., Pretzlaff et al., 2010; Solick & Barclay, 2006; Willis & Brigham, 2007). During the nonbreeding season without the burdens of nursing young, bats are relaxed from thermal constraints and frequently enter torpor to gain energetic benefits (e.g., Pretzlaff et al., 2010; Solick & Barclay, 2006); during this time, torpid bats tend to roost solitarily or in relatively small groups, suggesting that this behavior facilitated faster cooling and reduced the risk of disturbance from other aroused group members (Pretzlaff et al., 2010). However, for those species that do not enter torpor, individuals could gain benefits derived from social thermoregulation, particularly on cool days. In this regard, if physiological demands are the main factors driving sociality, we would predict that group size is more important than group composition, that group size is influenced by ambient temperature, and group composition is likely flexible. In addition to social thermoregulation, group living of bats confers several benefits, which include reduction in predation risk, increased vigilance, accessibility to mate, transfer of information about food and roosts, shared food, and potential cooperation and nepotism (Fenton et al., 1994; Kerth, 2008; Kerth & Reckardt, 2003; Safi & Kerth, 2007). Living in groups also incurs associated costs, such as increased predation risk, conspicuousness, increased competition for mates and resources, and increased disease transmission (Kerth, 2008). Sociality would be selected if the benefits of group living outweighs the costs in terms of inclusive fitness (Hamilton, 1964).

**FIGURE 1:**
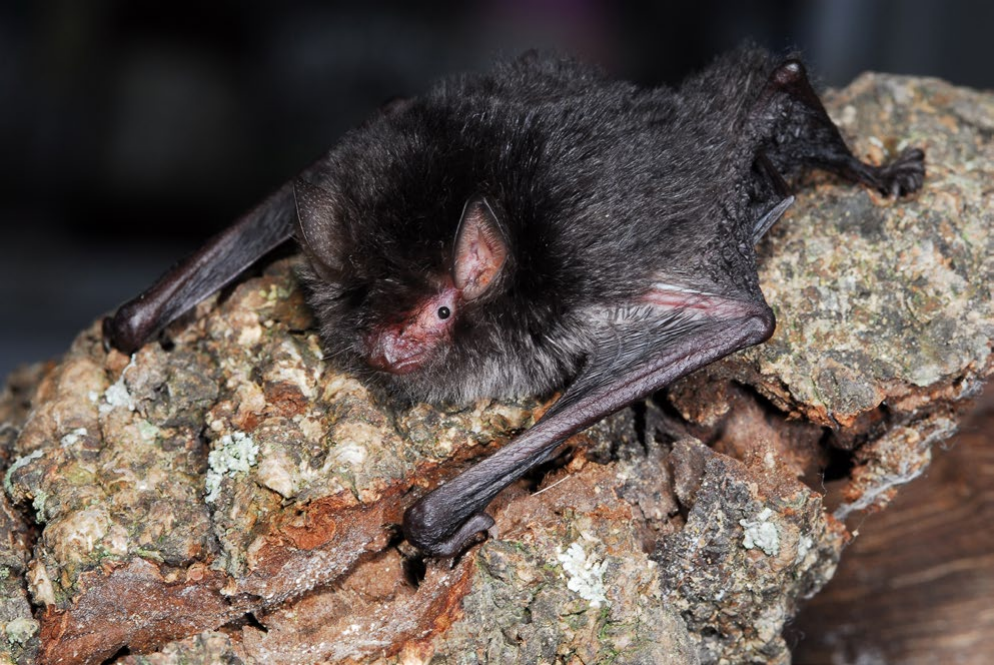
Dark woolly bat *Kerivoula furva*

The dark woolly bat *Kerivoula furva* (Chiroptera: Vespertilionidae; Figure 1) is a small (4–7 g) insectivorous bat that emits very broad bandwidths and extremely high frequency calls (Kuo et al., 2017). In subtropical Taiwan, it is widely distributed from low‐to‐middle elevational broadleaf evergreen forests. It has been reported to roost in furled leaves of banana (*Musa formosana*) plants solitarily or in small groups of 2–10 individuals (Kao et al., 2020; Kuo et al., 2017). Bats that use ephemeral roosts, such as furled banana leaves, are forced to find new roosts following foliation. Despite this, recent studies showed some bats that lived in roosts with short lifespans exhibited great group stability (e.g., Chaverri, 2010; Sagot & Stevens, 2012). *Kerivoula furva* could provide a good opportunity to study the potential mechanisms that are attributed to group formation and evolution of sociality in subtropical regions.

In this study, we described the roost characteristics and social organization of *K. furva* in subtropical Taiwan. We examined whether each of the three factors, roost availability, demographic traits, and social thermoregulation, contributed to group formation in *K. furva*. We tested the following three nonmutually exclusive hypotheses. (a) *K. furva* roost in groups because suitable roosts were limited (ecological constrains hypothesis). We predicted that the daily roost occupation rate would be high. (b) Groups of *K. furva* were actively aggregated by individuals from multiple generations (demographic traits hypothesis). We predicted that *K. furva* would exhibit natal fidelity and that social group members would have nonrandom associations. (c) *K. furva* roosted in groups to facilitate thermoregulation (thermoregulation hypothesis). We predicted that the groups of *K. furva* would be aggregated by random individuals; in addition, group size in both breeding and nonbreeding seasons would increase with decreasing ambient temperature.

## 2 MATERIALS AND METHODS

### 2.1 Study area

**FIGURE 2:**
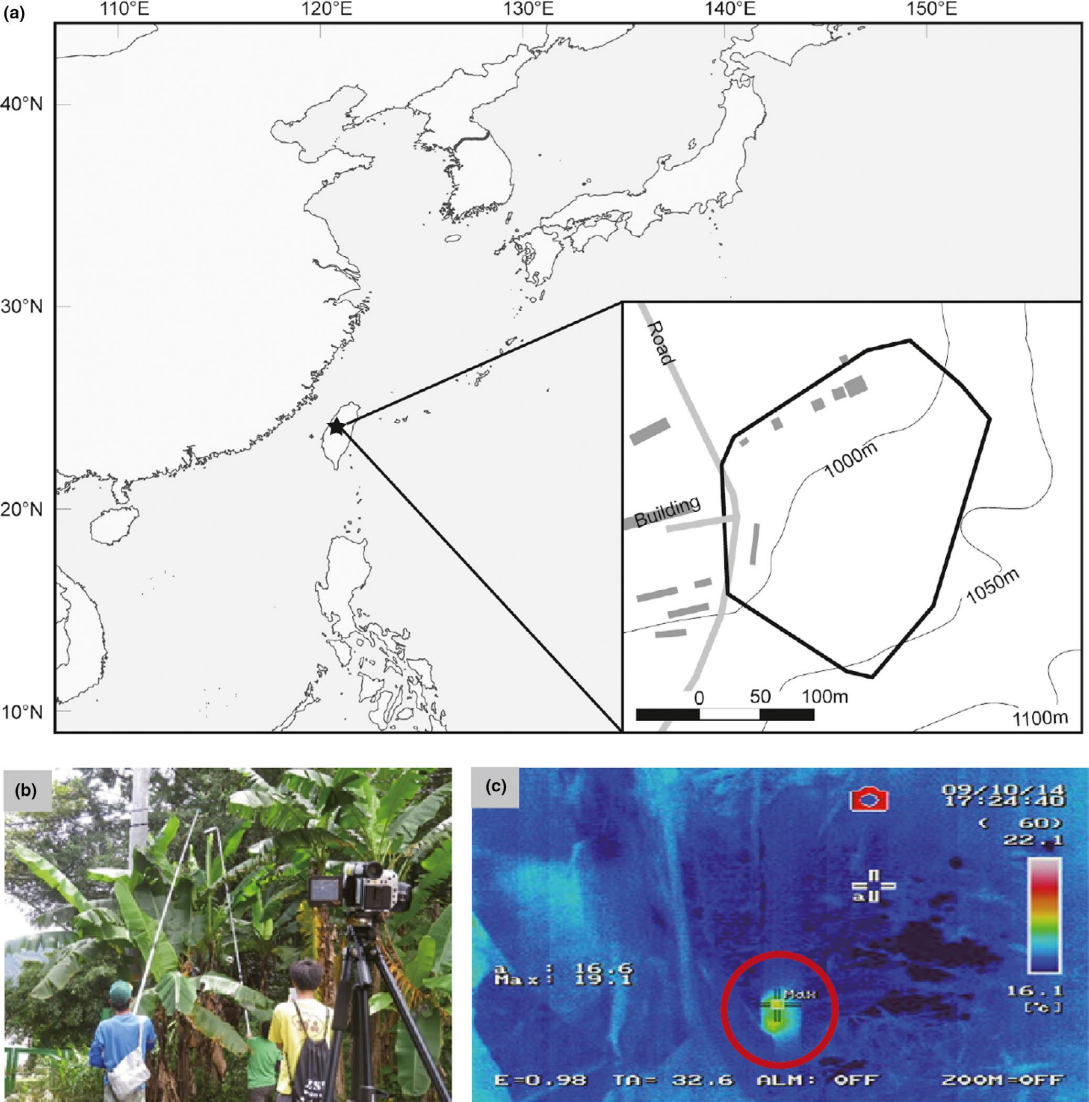
(a) Study area in Wushihkeng area, Taichung City, central Taiwan. (b) Banana (*Musa formosana*) grew on the edge of the forests. Researchers used a thermal image camera to detect the presence of bats, and they measured the characteristics of the roost. (c) Images from a thermal image camera of a banana furled leaf occupied by bats

We carried out this study at Wushihkeng, Taichung City, in central Taiwan (24°28′N, 120°95′E, Figure 2a). This area belonged to the Low Elevation Experimental Station of Endemic Species Research Institute, Taiwan. The banana plants were clumped in patches growing on the edge of plantations of Japanese cedar, *Cryptomeria japonica* (L.f.) D. Don (Figure 2b). The total area of our study site was 4.09 ha. Some small banana patches or individual plants that were distributed sparsely outside or study site were not surveyed on a regular basis and, thus, those data were excluded in our analysis. Air temperature and precipitation were recorded hourly by a meteorological station located at the study site (24°16′N, 120°56′E, 996 m a.s.l.). During the study period, the monthly average air temperature and precipitation, respectively, ranged between 12.6°C and 23.2°C and between 0 mm and 726 mm.

### 2.2 Roost characteristics and availability

From July 2014 to May 2016, we conducted 1–5 field censuses per month. In each census, 2–5 observers traversed the entire study area systematically and searched for the developing furled banana leaves during daytime. When a furled leaf was found, we marked the banana plant with a numbered plastic tag. We used a thermal image camera (NEC Infrared R300, Avionics Fukushima Co., Ltd) to detect presence of bats. If bats were present in the furled leaves, their body temperature created a bright spot under the thermal image camera (Figure 2b,c). However, the effectiveness of the thermal image camera decreased when bats entered deep torpor. If we were not able to determine whether bats were present by the thermal image camera, we used a sport camera (Hero3, GoPro Inc) to take photographs from the top of the furled leaf for confirmation. We attached the sport camera to a 6‐m pole and controlled it remotely by a cell phone (Figure 2b).

For each banana plant with a furled leaf, if reachable, we measured plant height, length of the curled leaf (hereafter leaf length), diameter of the curled leaf opening, and canopy coverage. We measured plant height and leaf length to the nearest 0.1 m by a 13‐m metered pole; we measured the diameter of the curled leaf opening to the nearest 0.1 cm by a homemade scale mounted on a 6‐m pole. We used the sport camera to take photographs to facilitate reading the scale. To measure the canopy coverage, we used the sport camera to take photographs of the canopy from the top of the furled leaf. The photographs were converted to black‐and‐white images by the ImageJ software (http://imagej.net/Downloads). We determined the canopy coverage as the percentage of image that was dark.

For each of the four roost characteristics, we first determined the range over which bats used a particular furled leaf. We then defined a furled leaf “suitable for roosting” if all four characteristics fell within the range of use. In a given day, we defined “roost availability” as the number of leaves suitable for roosting, and the “roost occupation rate” was the number of leaves that were occupied by bats divided by the number of leaves suitable for roosting.

To determine rate of unfurling, we selected unoccupied furled leaves randomly and measured the diameter of the opening twice a day at around 9 am and 4 pm. To examine whether the roosts provided thermal insulation function, we measured air temperatures inside (T_a‐in_) and outside (T_a‐out_) (<20 cm to the focal leaf) of unoccupied furled leaves using pairs of iButton (DS1921G Thermochron iButton, Maxim, USA) suspended in the air using fishing rod and line.

### 2.3 Roosting group size and composition

We captured bats in the late afternoon by covering the open end of the furled leaf with a cloth bag. We then cut the leaf from the base and removed the entire leaf. This approach allowed us to capture the whole group of bats without causing injury. Because furled leaves suitable for roosting last only a short time and only a small proportion of furled leaves were removed, we believed that removal of furled leaves had little effect on roost availability. Some of the furled leaves occupied by bats were not removed because they were too high or too difficult to reach. We used the thermal image camera to examine whether those unremoved roosts were repeatedly used by bats in subsequent days.

For each bat, we examined sex and age and assessed its reproductive condition. Males with swollen testicles and females with elongated nipples and signs of lactation were considered to be reproductive. Bats that had transparent metacarpal–phalangeal joints were aged as juveniles (Anthony, 1988), otherwise they were aged as adults. We measured body weight to the nearest 0.01g using a digital scale (SF‐718, Jiangyin Suofei Electronic Technology Co., Ltd) and measured forearm length and tarsus length to the nearest 0.1 mm with a digital caliper. We used a biopsy punch to take a tissue sample approximately 2 mm in diameter from the wing membrane for future genetic analysis. We band‐marked each bat with a numbered aluminum wing tag (2.9 mm, Porzana Ltd.) for individual identification. We did not band‐mark juveniles until July, when juveniles were volant or started to flip their wings. In addition, we assigned each bat an individual‐specific code; for example, 2014AM1 and 2014JM1, respectively, indicated the first adult male and first juvenile male captured in 2014. All bats from the same roost were released to the wild simultaneously after sunset.

We defined the bats in a given furled leaf as a “roosting group” even if there was only a single individual present; the “roosting group size” was the number of bats in a given leaf. We determined “group composition” using adults only. Each group was designated to one of eight categories: single‐male, single‐female, single‐male and multi‐female, multi‐male, multi‐female, singe‐male and single‐female, single‐female and multi‐male, and multi‐male and multi‐female.

All experimental procedures were approved by the Taichung City government (Issue No. 1,060,159,680) and conducted according to the ASAB/ABS Guidelines for the use of Animals in Research.

### 2.4 Association pattern

We used a simple ratio association index (SRI) (Cairns & Schwager, 1987; Flores et al., 2020; Hoppitt & Farine, 2018) to determine the pairwise roosting associations of bats based on repeated sampling data. To minimize the effects of potential parental care on association, we mainly used adults sampled ≥4 times for SRI analysis (Flores et al., 2020; Vonhof et al., 2004), except when bats were sampled three times as adults and one time as a volant juvenile. SRI is defined as the probability that a pair of individuals were found together in the same roost and calculated as X_AB_/(X_AB_ + X_A_ + X_B_ + X_O_), where X_AB_ is the number of censuses in which two individuals, A and B, roosted together. X_A_ and X_B_, respectively, are the number of censuses in which only individual A or B was found in a roost, and X_O_ is the number of censuses in which the two individuals were found in different roosts in a given day. We applied a hierarchical cluster analysis to describe social relations among individuals using the program SOCPROG ver. 2.8 (Whitehead, 2009). We used an arbitrary value of 0.1 as the threshold for separating social groups (Vonhof et al., 2004).

### 2.5 Data analysis

We presented characteristics of roosts and roosting group size as mean ± 1 standard deviation (*SD*). To test whether roosting group size was influenced by the characteristics of furled leaves or daily average air temperature, we first tested for collinearity among the characteristics and air temperature. We used a principal component analysis (PCA) to extract the variables that were correlated. We then performed a multiple linear regression to examine the relationships between the predictor variables and group size. The effect of air temperature on group size might differ according to reproductive status. We used Spearman correlations to test whether group size was influenced by daily average air temperature during the pregnancy/lactation season and the nonbreeding season. Bats gave birth between late May and mid‐June, and pups were able to fly at approximately 30 days old (Luo et al., 2020). We defined pregnancy/lactation season to be from May to mid‐July. We used a Mann–Whitney *U* test to compare the roosting group size between the pregnancy/lactation season and the nonbreeding season. We used a Pearson correlation to test the relationship between T_a‐in_ and T_a‐out_ and compared them with a paired Student's *t* test. We performed analysis of variance (ANOVA) or Kruskal–Wallis test, depending on the normality of the data, to test whether average SRI differed among the three sex classes (female–female, female–male, and male–male). Statistical significance was set at *p* < .05 for all tests.

## 3 RESULTS

### 3.1 Roost availability and roosting behavior

From July 2014 to May 2016, we conducted a total of 97 censuses. On 46 of the 97 censuses, we searched the entire study area and finished all the measurements within a single day; during the other 51 censuses, however, investigations were either interrupted by heavy rains, or we were unable to finish before sunset. Data from those incomplete censuses were included in roosting group size, group composition, and association pattern analyses, but they were excluded from analyses of daily roost availability and roost occupation rate.

We found a total of 168 furled leaves occupied by bats. *Kerivoula furva* was the only bat species found in furled leaves. All four leaf characteristics were measured completely for 118 banana plants. Average plant height, leaf length, diameter of the furled leaf opening, and canopy coverage of the occupied leaves were 4.43 ± 1.44 m (range = 1.39–7.19), 1.61 ± 0.46 m (range = 0.56–3.09), 11.9 ± 5.7 cm (range = 4.0–32.0), and 76.8 ± 16.5% (range = 28.9–99.5), respectively.

Daily roost availability ranged from 5 to 51 (mean 24.6 ± 9.7, *n* = 46) and increased with increasing daily average air temperature (*R* ^2^ = 0.39, *p* = .001). The daily number of occupied roosts averaged 1.6 ± 1.6 (range = 0–6) with 0–21 bats found per day. The daily roost occupation rate was 0%–26.1% with an average of 6.7 ± 6.8% (*n* = 46).

We observed the unfurling rate of six leaves in winter (5–13 February 2015) and 15 leaves in summer (4–6 July 2015 and 22–25 July 2015). The duration of the furled leaves suitable for roosting (opening diameter between 4.0 cm and 32.0 cm) lasted <31 hr in summer, but was ≥8 days in winter (Figure S1).

We simultaneously measured T_a‐in_ and T_a‐out_ of three unoccupied furled leaves. In two of them, we measured for 24 hr from 17:00 on 10 October 2014 to 17:00 on 11 October 2014. We measured the third leaf from 11:00 to 16:00 on 11 February 2015. In general, the difference between T_a‐in_ and T_a‐out_ was small (<1°C, Figure S2). T_a‐in_ was highly correlated with T_a‐out_ (all *r* > 0.97) and did not differ significantly from it in all three comparisons.

We observed 32 roosts in July and August (summer) and eight roosts in January and February (winter) to determine whether bats repeatedly used the same roosts on subsequent days. With the exception of one roost that was used for three consecutive days in January 2015, all other roosts were used for only one day.

### 3.2 Roosting group size and group composition

**FIGURE 3:**
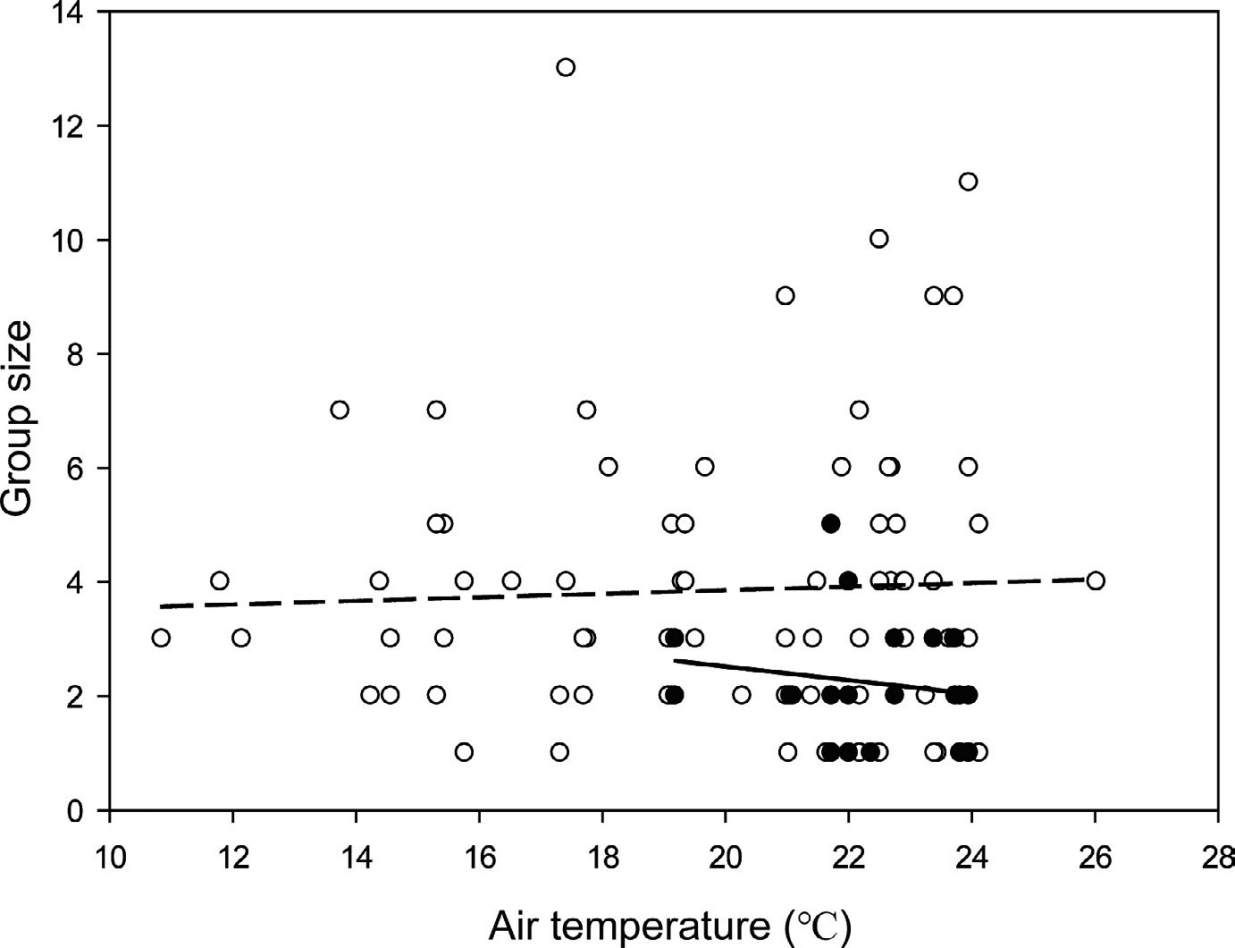
The relationship between group size of *Kerivoula furva* and air temperature between July 2014 and May 2016. Filled (solid line) and empty circles (dash line), respectively, indicate data collected during pregnancy/lactation season (from May to mid‐July) and nonbreeding season. Solid line *y* = 4.92–0.12*x*, *r* = 0.16, *p* = .49, *n* = 21; dashed line *y* = 3.23 + 0.03*x*, *r* = 0.04, *p* = .69, *n* = 83

We removed 110 furled leaves, but on six occasions at least one bat escaped during the operation. Although the number of bats that escaped was observed, to be conservative our analysis only included those 104 groups in which all bats were captured. Size of roosting groups ranged from 1 to 13 with a median of 3 and an average of 3.8 ± 2.4. When we removed the juveniles from analysis, the roosting group size ranged from 1 to 13 with a median of 3 and an average of 3.3 ± 2.0 (*n* = 104, Figure 3). Among the characteristics of roosts and temperature, we found that leaf length and diameter of the furled leaf were correlated (Pearson correlation coefficient, *r* = 0.896, *p* < .001). We used a PCA with varimax rotation to remove the correlations in these two variables. The PCA extracted a principal component that can explain at least 94% of the variation. Overall, the predictive variables had no significant effects on group size (*p* = .063, Table S1). Roosting group size did not vary with air temperature in either the pregnancy/lactation season or the nonbreeding season (Figure 3). Roosting group size during the breeding season (2.2 ± 1.0, *n* = 21) was significantly smaller than that during nonbreeding season (3.9 ± 2.4, *n* = 83, Mann–Whitney *U* test: *U* = 483.5, *p* = .001).

The composition of the 104 groups was highly variable, from entirely female to entirely male. When only adults were considered, 45.2% of the groups were composed of multiple females, followed by single‐male and multi‐female (17.3%) and multi‐male and multi‐female (10.6%) (Table 1). Multi‐female groups were the most common group in all months except November. During the pregnancy/lactation period, the vast majority of reproductive females roosted with pups, but males primarily roosted separately from females either singly or in small bachelor groups.

**TABLE 1:** Group composition of adult *Kerivoula furva* from July 2014 to May 2016 in central Taiwan

Month	Single‐male	Multi‐male	Single‐female	Multi‐female	Single‐male/Single‐female	Single‐male/Multi‐female	Multi‐male/Single‐female	Multi‐male/Multi‐female
January	0	0	0	0	0	0	0	1
February	0	1	0	5	0	2	0	1
March	0	1	0	5	1	2	0	1
April	0	2	1	2	0	0	0	3
May	0	1	1	11	0	1	0	0
June	0	1	0	3	0	0	0	0
July	1	1	0	7	0	0	2	1
August	2	1	1	2	2	4	2	2
September	2	1	1	6	0	2	0	0
October	0	1	0	3	0	2	0	0
November	0	0	0	1	0	3	0	2
December	0	0	1	2	1	2	0	0
Total (%)	5 (4.8)	10 (9.6)	5 (4.8)	47 (45.2)	4 (3.8)	18 (17.3)	4 (3.8)	11 (10.6)

Note

The values indicate the number of bat groups found.

### 3.3 Association pattern and natal fidelity

**FIGURE 4:**
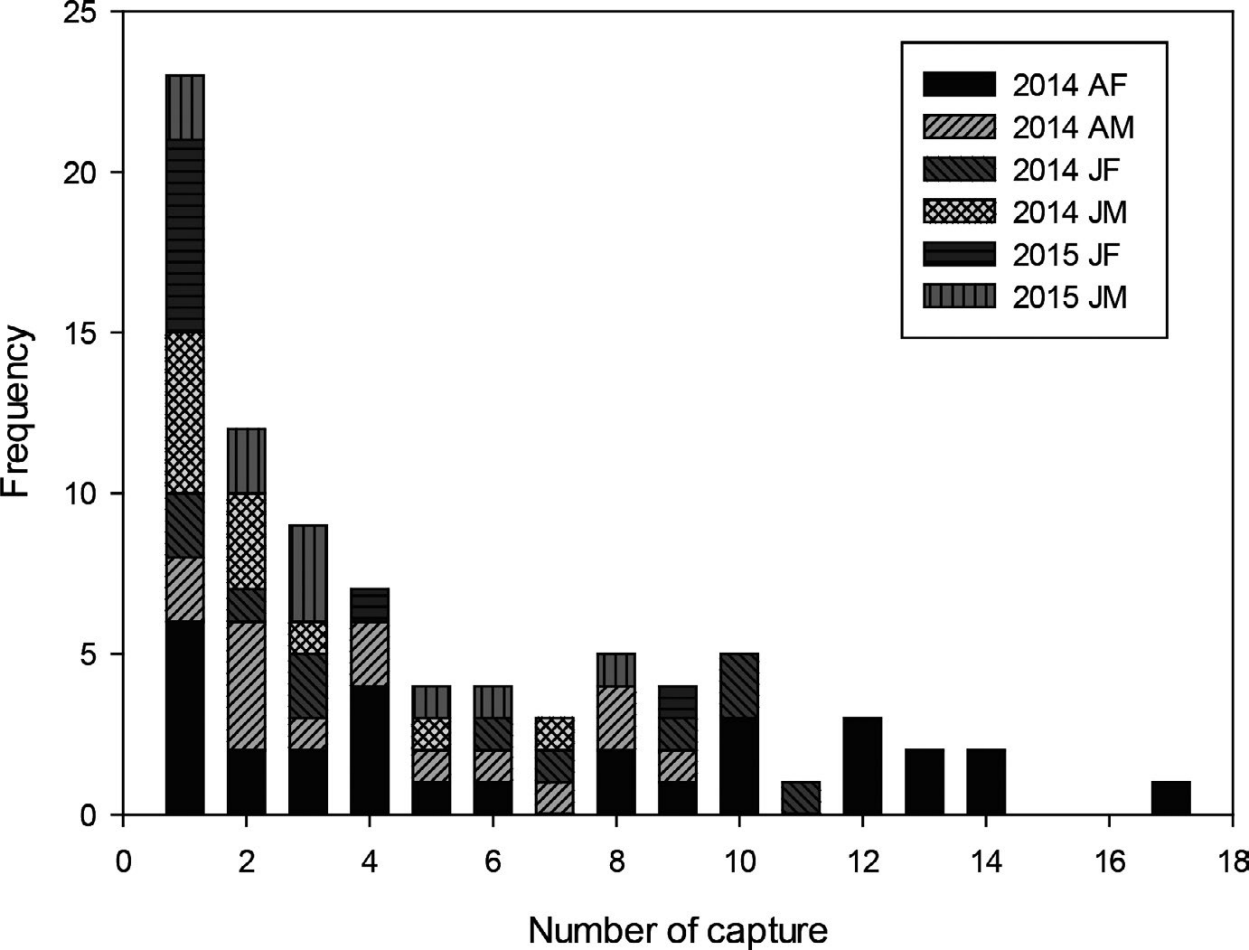
The number of captures of 85 *Kerimoula furva* between July 2014 and May 2016 in central Taiwan. AF, AM, JF, and FM indicate the bat was determined as adult female, adult male, juvenile female, or juvenile male, respectively, at first capture

**FIGURE 5:**
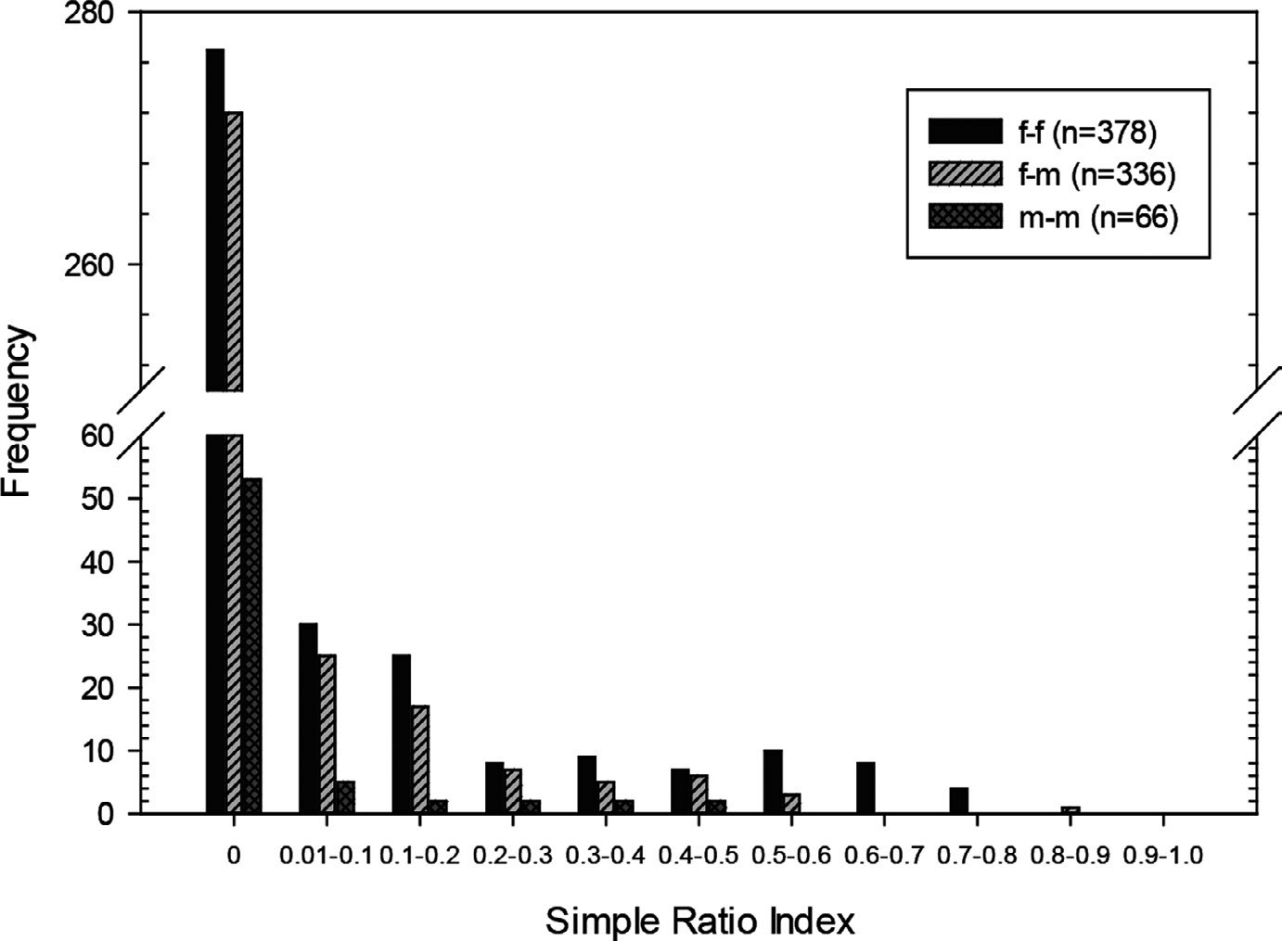
Simple ratio index of pairs of *Kerivoula furva* in central Taiwan between July 2014 and May 2016. Only bats that were adults and sampled ≥4 times were used for analysis. The letter f and m indicate female and male, respectively

We identified a total of 85 individuals during the study period, which included 15 adult males, 30 adult females, 11 juvenile males and 11 juvenile females born in 2014, and 10 juvenile males and eight juvenile females born in 2015. During the study period, these 85 individuals were captured a total of 393 times with 1–17 captures per individual (Figure 4). All bats that were sampled ≥10 times were females. Of the 85 bats, 28 females and 12 males were used for SRI analysis. The observed dyad SRIs of those 40 individuals ranged from 0 to 0.83 (Table S2), with an average of 0.05 ± 0.14 (*n* = 780). The average SRI of female–female pairs, male–female pairs, and male–male pairs, respectively, was 0.07 ± 0.16 (range = 0–0.77, *n* = 378), 0.04 ± 0.11 (range = 0–0.83, *n* = 336), and 0.04 ± 0.10 (range = 0–0.45, *n* = 66) (Figure 5). Average SRI was significantly different among the three sex classes (Kruskal–Wallis test, H = 7.447, *p* = .024). The average SRI of female–female pairs was significantly higher than that of male–female pairs (Mann–Whitney *U* test: *U* = 58,145.5, *p* < .01), but no significant differences were found in other pairwise comparisons.

**FIGURE 6:**
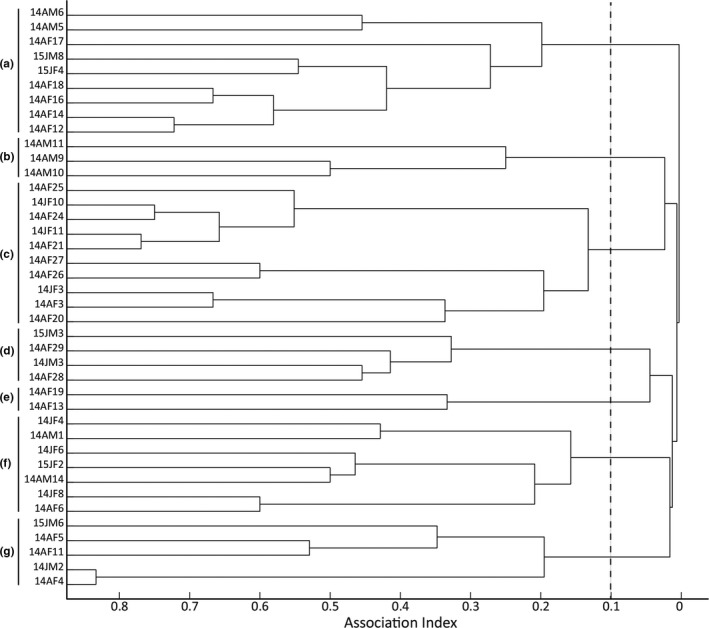
Cluster diagram of 40 individuals of *Kerivoula furva* using SocProg2.8 based on the dyad Simple Ratio Index. Bats were sampled at least four times between July 2014 and May 2016. Individual code AM: adult male. AF: adult female. JM: juvenile male JF: juvenile female. The number before the individual code refers to the year that bat was first capture. The 40 individuals were divided into seven social groups (A‐G)

Based on the cluster analysis, 40 individuals were separated into seven social groups with 2–10 individuals in each social group (Figure 6). The cophenetic correlation coefficient was 0.957 with clustering using average linkage. Within each social group, SRI averaged between 0.28 and 0.62. The composition of social groups included one all‐male group, four all‐female groups, and two mixed‐sex groups (Figure 6).

Of the 11 males and 11 females born in 2014, one male and six females stayed in their natal groups for ≥20 months (last captured between March and May 2016); one male and one female stayed in their natal groups ≥7 months (last capture in February 2015); nine males and four females were never recaptured after 1 January 2015.

## 4 DISCUSSION

### 4.1 Association pattern and demographic traits hypothesis

*Kerivoula furva* frequently switched day roosts, especially in summer during which roosts persisted for only 1 day. Despite that daily suitable leaves were often abundant, *K. furva* actively aggregated and maintained relatively stable social groups throughout the years. This indicated that *K. furva* was more likely to exhibit social preferences than shared interests in resources (Whitehead, 2008). In addition, only two of the eleven males born in 2014 stayed in their natal groups ≥6 months, but more than half of the females born in 2014 remained in their natal groups for ≥20 months, which suggested a female‐biased natal philopatry in *K. furva*. Consequently, social groups were composed of individuals from multiple generations. Taken together, our results supported the demographic traits hypothesis that longevity combined with female philopatry promoted sociality in *K. furva*. Bats generally have relatively long lifespans compared with similar‐sized mammals (Wilkinson & Adams, 2019); some species have been reported to live for ≥30 years (Wilkinson & South, 2002). In our study, one of the bats was caught and tagged in February 2011 as an adult (H.‐C. Cheng, personal communication); this individual was last captured in May 2016. This indicates that *K. furva* can live at least 6 years.

The subtropical *K. furva* formed distinct social groups, and those groups were relatively stable year‐round. The stable social groups have been reported for some tropical bats (e.g., Chaverri, 2010; Kerth, 2008; Sagot, 2016; Vonhof et al., 2004). In contrast to other social bats, the average SRI values of *K. furva* (0.04–0.07) are similar to that of *Phyllostomus hastatus* (0.059), *Desmodus rotundus* (0.021–0.061), and *Myotis septentrionalis* (0.057), but are lower than that of *S. bilineata* (0.107–0.112), *T. tricolor* (0.099–0.136), *Rhynchonycteris naso* (0.126–0.167), and *Myotis bechsteinii* (0.262) (reviewed by Wilkinson et al., 2019). Among the 40 individuals where we determined the association index, many pairs showed a SRI value of ≥0.5, and their relationships were maintained for ≥1 year. The strong social bond was especially significant for female–female pairs. The highest SRI (0.83), however, was observed between a female (14AF4) and a male (14JM2) during 7 months (from 1 August 2014 to 27 February 2015). We suspect the relationship between this pair was mother and son. This nonrandom association pattern was also found in other bats that roost in furled leaves, such as Spix's disk‐winged bats (*T. tricolor*). In *T. tricolor*, the majority of dyads remained together for up to 100 days and 40% of the dyads maintained associations for up to 420 days (Vonhof & Fenton, 2004). Other studies showed that groups of *T. tricolor* were composed mainly of philopatric individuals that were closely related (Buchalski et al., 2014; Wilkinson et al., 2019), and group composition remained unchanged for up to 22 months (Chaverri, 2010). Whether the social group members of *K. furva* with strong social bonds were related warrants further investigation using genetic analysis.

The nonrandom associations in *K. furva* suggested that they were able to identify and relocate social group members and to maintain social bonds (Chaverri et al., 2018). Bats use a variety of communication tools, such as acoustic (e.g., Arnold & Wilkinson, 2011; Chaverri & Gillam, 2016), olfactory (Englert & Greene, 2009; Safi & Kerth, 2003), and tactile signals (Kerth, 2006). *Thyroptera tricolor* uses social calls with distinctive features to communicate (Gillam & Chaverri, 2012). Whether *K. furva* uses social call to distinguish social group members from nonsocial group members remains untested. However, echolocation calls might also have a communication function in *K. furva* (Kao et al., 2020; Luo et al., 2020). Future study could focus on what cues (e.g., social call, echolocation call, odor, or visual) that *K. furva* uses to recognize group members.

### 4.2 Roosting ecology and roost availability hypothesis

The daily roost occupation rate in our study area was generally low, with an average of 6.7%, which suggested that a shortage of suitable roosts (ecological constraints) was not the main cause of group formation in *K. furva*. A low roost occupation rate was also found in other bats that roost in furled leaves; for example, the occupation rate was 5.7%–12% in *T. tricolor* (Vonhof & Fenton, 2004) and 15%–35% in *Neoromicia nanus* (*Pipistrellus nanus* in LaVal & LaVal, 1977; Happold & Happold, 1996; van der Merwe & Stirnemann, 2009). This suggests that for the bats that use furled leaves as day roosts group formation is not driven by insufficient roosts.

An individual furled leaf was suitable for *K. furva* to roost for only 1 day in summer and, therefore, *K. furva* was forced to switch roosts on a daily basis. Switching roosts frequently was energetically costly, especially when females had to carry nonvolant young to a new roost. It also increased the risks of exposure to nocturnal predators. Why bats chose to use such an ephemeral roost is of interest. Happold and Happold (1990) pointed out that furled banana leaves have three advantages: They are humid internally, abundant throughout the year, and interspecific competition for these roost locations is limited. In our study area, roosts were abundant and no roost competitors were found. Although we have no data on relative humidity inside the furled leaves, a previous study showed that the relative humidity within the furled leaf of banana (*Musa acuminate*) was always ≥80% (van der Merwe & Stirnemann, 2009). Nevertheless, here we propose an additional plausible explanation. In our study area, banana plants primarily grew on the edge of forests and were exposed to sunlight to some degree depending on the location of the banana plants. Because the banana furled leaves provided little or no insulation against air temperature (Figure S2), reproductive females may have selected roosts that were more exposed to sunlight to warm up rapidly. This warm roost may have facilitated reproductive females to reduce energetic costs of maintaining normothermia. During the nonbreeding season, based on our observations on the thermal camera, we found that *K. furva* frequently reduced their body temperatures, which suggested they entered torpor. *Kerivoula furva* may have selected roosts that were cool in the morning, but which received direct sunlight in late afternoon to gain an energetic benefit from passive rewarming. We observed that roost temperatures were elevated in the late afternoon of 9 October 2014 (e.g., Figure S2b,c), which supported our suggestion. During a cold winter, *K. furva* might enter multiple‐day torpor. Bats may have selected roosts that remained cool all day to avoid unnecessary passive arousals. Further investigation could explore the seasonal variation in torpor use by *K. furva* and its relation to roosting behaviors.

Of the 85 individuals recorded, 23 were never recaptured. We assumed some juveniles, especially males, left the study area due to natal dispersal. Some individuals, however, may have roosted regularly in banana plants outside our study range, but visited the study site occasionally. Another possibility is that *K. furva* used different types of roosts, such as bamboo internodes (Kuo et al., 2017). An investigation using radio telemetry might help to answer this question.

### 4.3 Size of roosting groups and the thermoregulation hypothesis

In this study, the size of roosting groups did not vary with temperature, and bats maintained long‐term, nonrandom associations, which indicated that group composition was more important than group size. Our results did not lend sufficient support to the social thermoregulation hypothesis. During the nonbreeding season, *K. furva* was likely to enter torpor frequently. In *Myotis bechsteinii*, torpid bats roosted solitarily or in relatively small groups, which suggested that they gained energetic benefits of fast cooling or avoided disturbances from other group members (Pretzlaff et al., 2010). For *K. furva*, social relationships during the nonbreeding seasons were likely shaped by individual preferences to associate with kin. By clustering together, group members might profit each other due to accelerated arousal in late afternoon.

During the pregnancy/lactation season, reproductive females actively aggregated, but group size was also independent of air temperature. Communal roosting may have facilitated reproductive females to meet physiological demands (e.g., Pretzlaff et al., 2010; Russo et al., 2017; Willis & Brigham, 2007), and this is especially important for species that us roosts that are poorly insulated against temperature (Russo et al., 2017). However, at our study site temperatures were warm throughout the breeding season. Social thermoregulation might not be important for *K. furva* and other bats that live in warm places (Kerth, 2008). This was supported by the result that *K. furva* maintained a relatively small roosting group size during the breeding season. Thus, although reproductive bats could benefit from group living, our results suggested that social thermoregulation was unlikely the main cause that drove group formation in *K. furva*.

The roosting group composition of *K. furva* was complex but similar to other bat species that roosted in furled leaves (Happold & Happold, 1990, 1996; Vonhof & Fenton, 2004). van der Merwe and Stirnemann (2009) reported that the composition of roosting groups of *P. nanus* was closely related to the reproductive cycle. Similar to the study of Bernard et al. (1997) and van der Merwe and Stirnemann (2009) on *P. nanus*, we found that males of *K. furva* roosted separately from the female groups either solitarily or in a small bachelor groups during the lactation period (June and early July), but they joined the female groups more often right after the lactation period, suggesting to seek the opportunity for mating.

## 5 CONCLUSION

To the best of our knowledge, our study was the first detailed study that describes social organization of subtropical bats in the Oriental Region. Although they changed roosts frequently, *K. furva* exhibited nonrandom associations and formed moderately stable social groups throughout the year. Because social groups of *K. furva* were composed of overlapping generations, this supported the demographic traits hypothesis for the cause of group formation. However, we did not find evidence to support the ecological constrains hypothesis and thermoregulation hypothesis in *K. furva*. Our study not only adds to the literature on the roosting ecology of a little known bat living in ephemeral roosts, but also provides valuable insights into the understanding of the evolution of sociality in bats.

## CONFLICT OF INTEREST

None declared.

## AUTHOR CONTRIBUTIONS

**Chia‐Wei Hsu:** Data curation (lead); formal analysis (equal); investigation (lead); methodology (equal); visualization (equal); writing–original draft (equal). **Mei‐Ting Kao:** Data curation (supporting); formal analysis (equal); visualization (equal); writing–original draft (supporting); writing–review and editing (supporting). **Cheng‐Han Chou:** Conceptualization (equal); investigation (supporting); methodology (equal); writing–review and editing (supporting). **Hsi‐Chi Cheng:** Conceptualization (equal); methodology (equal); project administration (equal); resources (equal); writing–review and editing (supporting). **Jian‐Nan Liu:** Conceptualization (equal); formal analysis (equal); funding acquisition (lead); investigation (supporting); methodology (equal); project administration (equal); resources (equal); supervision (lead); validation (lead); visualization (equal); writing–original draft (supporting); writing–review and editing (lead).

## Supporting information

Fig S1

Fig S2

Table S1

Table S2

## Data Availability

Data are available at Tables S2.
