# Supplementary figures and images for: Tests of hypotheses for group formation in the subtropical leaf‐dwelling bat, Kerivoula furva
Source: Ecol Evol. 2021 Apr 3;11(11):6730–41. doi: 10.1002/ece3.7524 (PMC8207392; doi:10.1002/ece3.7524)

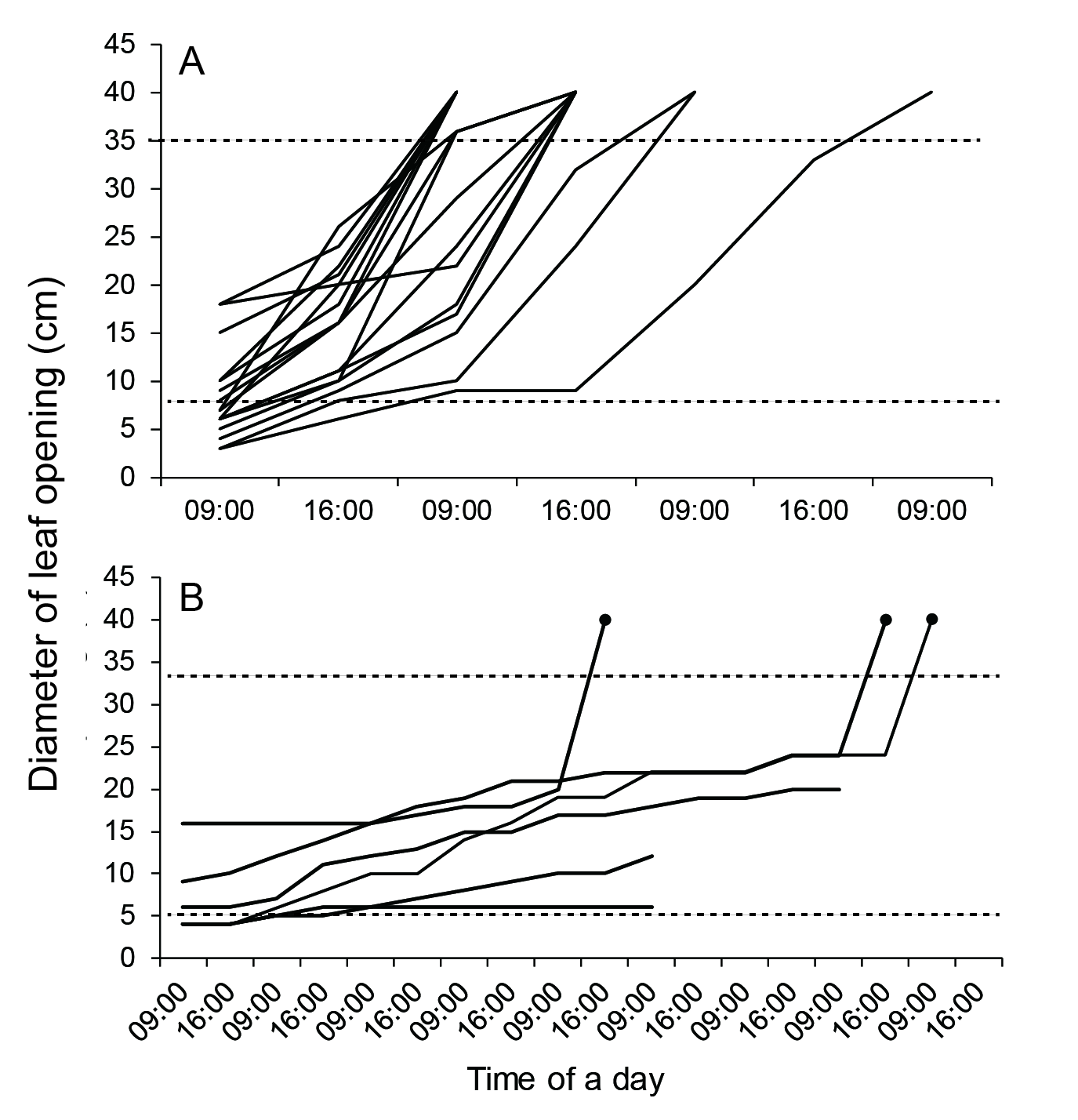

Supplement: Supplementary file 1 — Fig S1 [file ECE3-11-6730-s004.tif]
