# Supplementary material for: Tests of hypotheses for group formation in the subtropical leaf‐dwelling bat, Kerivoula furva
Source: Ecol Evol. 2021 Apr 3;11(11):6730–41. doi: 10.1002/ece3.7524 (PMC8207392; doi:10.1002/ece3.7524)
Supplement: Supplementary file 2 — Fig S2 [file ECE3-11-6730-s002.docx]

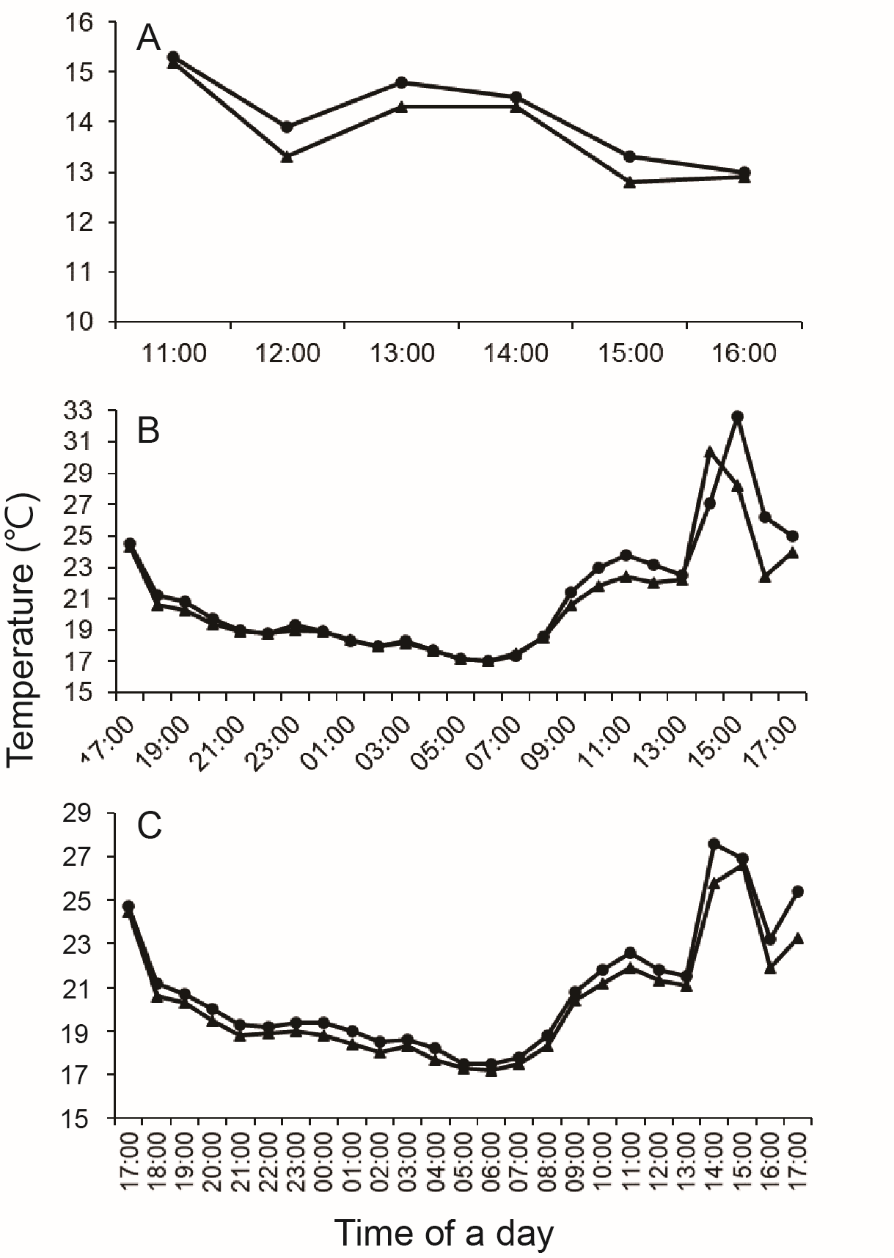


**Figure S2** Temperatures inside (circle) and outside (triangle) of three furled leaves of banana (*Musa formosana*) recorded by a pair of iButton. The data were collected on (A) 2015/2/11, (B) 2014/10/9-10, and (C) 2014/10/9-10
