# Supplementary material for: Tests of hypotheses for group formation in the subtropical leaf‐dwelling bat, Kerivoula furva
Source: Ecol Evol. 2021 Apr 3;11(11):6730–41. doi: 10.1002/ece3.7524 (PMC8207392; doi:10.1002/ece3.7524)
Supplement: Supplementary file 3 — Table S1 [file ECE3-11-6730-s003.docx]

**TABLE S1** A multiple linear regression was used to test whether the roosting group size of *Kerivoula furva* was influenced by the characteristics of banana (*Musa formosana*) furled leaves and daily average air temperature

| Model | Sum of Squares | df | Mean square | F | p-value |
| --- | --- | --- | --- | --- | --- |
| Regression | 54.97 | 4 | 13.743 | 2.390 | 0.063 |
| Residual | 298.96 | 52 | 5.749 |  |  |
| Total | 353.93 | 56 |  |  |  |
