# Supplementary material for: Tests of hypotheses for group formation in the subtropical leaf‐dwelling bat, Kerivoula furva
Source: Ecol Evol. 2021 Apr 3;11(11):6730–41. doi: 10.1002/ece3.7524 (PMC8207392; doi:10.1002/ece3.7524)
Supplement: Supplementary file 4 — Table S2 [file ECE3-11-6730-s001.docx]

**TABLE S2** Dyad associate indices of 40 individuals of *Kerivoula furva* in central Taiwan

|  | **14AF11** | **14AF12** | **14AF13** | **14AF14** | **14AF16** | **14AF17** | **14AF18** | **14AF19** | **14AF20** | **14AF21** | **14AF24** | **14AF25** | **14AF26** | **14AF27** | **14AF28** | **14AF29** | **14AF3** | **14AF4** | **14AF5** | **14AF6** |
| --- | --- | --- | --- | --- | --- | --- | --- | --- | --- | --- | --- | --- | --- | --- | --- | --- | --- | --- | --- | --- |
| 14AF11 |  | 0 | 0 | 0 | 0 | 0 | 0 | 0.06 | 0 | 0 | 0 | 0 | 0 | 0 | 0.05 | 0 | 0.05 | 0.27 | 0.5 | 0.05 |
| 14AF12 |  |  | 0 | 0.72 | 0.58 | 0.39 | 0.53 | 0 | 0 | 0 | 0.04 | 0 | 0 | 0 | 0 | 0 | 0 | 0 | 0 | 0 |
| 14AF13 |  |  |  | 0 | 0 | 0 | 0 | 0.4 | 0 | 0 | 0 | 0 | 0 | 0 | 0 | 0 | 0.27 | 0 | 0 | 0 |
| 14AF14 |  |  |  |  | 0.69 | 0.47 | 0.53 | 0 | 0 | 0 | 0.04 | 0 | 0 | 0 | 0 | 0 | 0 | 0 | 0 | 0 |
| 14AF16 |  |  |  |  |  | 0.31 | 0.67 | 0 | 0 | 0 | 0.05 | 0 | 0 | 0 | 0 | 0 | 0 | 0 | 0 | 0 |
| 14AF17 |  |  |  |  |  |  | 0.33 | 0 | 0 | 0 | 0 | 0 | 0 | 0 | 0 | 0 | 0 | 0 | 0 | 0 |
| 14AF18 |  |  |  |  |  |  |  | 0 | 0 | 0 | 0.05 | 0 | 0 | 0 | 0 | 0 | 0 | 0 | 0 | 0 |
| 14AF19 |  |  |  |  |  |  |  |  | 0 | 0 | 0 | 0 | 0 | 0 | 0 | 0 | 0 | 0 | 0 | 0 |
| 14AF20 |  |  |  |  |  |  |  |  |  | 0.23 | 0.14 | 0.17 | 0.33 | 0.33 | 0 | 0 | 0.27 | 0 | 0 | 0 |
| 14AF21 |  |  |  |  |  |  |  |  |  |  | 0.64 | 0.5 | 0.07 | 0.14 | 0 | 0 | 0.17 | 0 | 0.05 | 0 |
| 14AF24 |  |  |  |  |  |  |  |  |  |  |  | 0.54 | 0.14 | 0.14 | 0 | 0 | 0.11 | 0 | 0.05 | 0 |
| 14AF25 |  |  |  |  |  |  |  |  |  |  |  |  | 0.08 | 0.17 | 0 | 0 | 0.11 | 0 | 0 | 0 |
| 14AF26 |  |  |  |  |  |  |  |  |  |  |  |  |  | 0.6 | 0 | 0 | 0.08 | 0 | 0 | 0 |
| 14AF27 |  |  |  |  |  |  |  |  |  |  |  |  |  |  | 0 | 0 | 0.08 | 0 | 0 | 0 |
| 14AF28 |  |  |  |  |  |  |  |  |  |  |  |  |  |  |  | 0.4 | 0 | 0 | 0.05 | 0 |
| 14AF29 |  |  |  |  |  |  |  |  |  |  |  |  |  |  |  |  | 0 | 0 | 0 | 0 |
| 14AF3 |  |  |  |  |  |  |  |  |  |  |  |  |  |  |  |  |  | 0 | 0 | 0 |
| 14AF4 |  |  |  |  |  |  |  |  |  |  |  |  |  |  |  |  |  |  | 0.29 | 0 |
| 14AF5 |  |  |  |  |  |  |  |  |  |  |  |  |  |  |  |  |  |  |  | 0 |
| 14AF6 |  |  |  |  |  |  |  |  |  |  |  |  |  |  |  |  |  |  |  |  |
| 14AM1 |  |  |  |  |  |  |  |  |  |  |  |  |  |  |  |  |  |  |  |  |
| 14AM10 |  |  |  |  |  |  |  |  |  |  |  |  |  |  |  |  |  |  |  |  |
| 14AM11 |  |  |  |  |  |  |  |  |  |  |  |  |  |  |  |  |  |  |  |  |
| 14AM14 |  |  |  |  |  |  |  |  |  |  |  |  |  |  |  |  |  |  |  |  |
| 14AM5 |  |  |  |  |  |  |  |  |  |  |  |  |  |  |  |  |  |  |  |  |
| 14AM6 |  |  |  |  |  |  |  |  |  |  |  |  |  |  |  |  |  |  |  |  |
| 14AM9 |  |  |  |  |  |  |  |  |  |  |  |  |  |  |  |  |  |  |  |  |
| 14JF10 |  |  |  |  |  |  |  |  |  |  |  |  |  |  |  |  |  |  |  |  |
| 14JF11 |  |  |  |  |  |  |  |  |  |  |  |  |  |  |  |  |  |  |  |  |
| 14JF3 |  |  |  |  |  |  |  |  |  |  |  |  |  |  |  |  |  |  |  |  |
| 14JF4 |  |  |  |  |  |  |  |  |  |  |  |  |  |  |  |  |  |  |  |  |
| 14JF6 |  |  |  |  |  |  |  |  |  |  |  |  |  |  |  |  |  |  |  |  |
| 14JF8 |  |  |  |  |  |  |  |  |  |  |  |  |  |  |  |  |  |  |  |  |
| 14JM2 |  |  |  |  |  |  |  |  |  |  |  |  |  |  |  |  |  |  |  |  |
| 14JM3 |  |  |  |  |  |  |  |  |  |  |  |  |  |  |  |  |  |  |  |  |
| 15JF2 |  |  |  |  |  |  |  |  |  |  |  |  |  |  |  |  |  |  |  |  |
| 15JF4 |  |  |  |  |  |  |  |  |  |  |  |  |  |  |  |  |  |  |  |  |
| 15JM3 |  |  |  |  |  |  |  |  |  |  |  |  |  |  |  |  |  |  |  |  |
| 15JM6 |  |  |  |  |  |  |  |  |  |  |  |  |  |  |  |  |  |  |  |  |
| 15JM8 |  |  |  |  |  |  |  |  |  |  |  |  |  |  |  |  |  |  |  |  |

**TABLE S2** *Cont.*

|  | **14AM1** | **14AM10** | **14AM11** | **14AM14** | **14AM5** | **14AM6** | **14AM9** | **14JF10** | **14JF11** | **14JF3** | **14JF4** | **14JF6** | **14JF8** | **14JM2** | **14JM3** | **15JF2** | **15JF4** | **15JM3** | **15JM6** | **15JM8** |
| --- | --- | --- | --- | --- | --- | --- | --- | --- | --- | --- | --- | --- | --- | --- | --- | --- | --- | --- | --- | --- |
| 14AF11 | 0 | 0 | 0 | 0.06 | 0 | 0 | 0 | 0 | 0 | 0 | 0.11 | 0.06 | 0.05 | 0.25 | 0 | 0 | 0 | 0.06 | 0.25 | 0 |
| 14AF12 | 0 | 0 | 0 | 0 | 0.25 | 0.16 | 0 | 0 | 0 | 0 | 0 | 0 | 0 | 0 | 0 | 0 | 0.44 | 0 | 0 | 0.39 |
| 14AF13 | 0.08 | 0 | 0 | 0 | 0 | 0 | 0.07 | 0 | 0 | 0.36 | 0.07 | 0 | 0.06 | 0 | 0 | 0 | 0 | 0 | 0 | 0 |
| 14AF14 | 0 | 0 | 0 | 0 | 0.22 | 0.18 | 0 | 0 | 0 | 0 | 0 | 0 | 0 | 0 | 0 | 0 | 0.35 | 0 | 0 | 0.38 |
| 14AF16 | 0 | 0 | 0 | 0 | 0.31 | 0.19 | 0 | 0 | 0 | 0 | 0 | 0 | 0 | 0 | 0 | 0 | 0.47 | 0 | 0 | 0.4 |
| 14AF17 | 0 | 0 | 0 | 0 | 0.14 | 0.18 | 0 | 0 | 0 | 0 | 0 | 0 | 0 | 0 | 0 | 0 | 0.06 | 0 | 0 | 0.07 |
| 14AF18 | 0 | 0 | 0 | 0 | 0.33 | 0.2 | 0 | 0 | 0 | 0 | 0 | 0 | 0 | 0 | 0 | 0 | 0.5 | 0 | 0 | 0.43 |
| 14AF19 | 0.13 | 0 | 0 | 0 | 0 | 0 | 0.1 | 0 | 0 | 0.07 | 0.09 | 0 | 0.08 | 0 | 0 | 0 | 0 | 0 | 0 | 0 |
| 14AF20 | 0 | 0 | 0 | 0 | 0 | 0 | 0.11 | 0.18 | 0.15 | 0.4 | 0 | 0 | 0 | 0 | 0 | 0 | 0 | 0 | 0 | 0 |
| 14AF21 | 0 | 0 | 0 | 0 | 0 | 0 | 0.06 | 0.75 | 0.77 | 0.16 | 0 | 0 | 0 | 0 | 0 | 0 | 0 | 0 | 0.06 | 0 |
| 14AF24 | 0 | 0 | 0 | 0 | 0.05 | 0.06 | 0.06 | 0.75 | 0.57 | 0.11 | 0 | 0 | 0 | 0 | 0 | 0 | 0.06 | 0 | 0.06 | 0.06 |
| 14AF25 | 0 | 0 | 0 | 0 | 0 | 0 | 0.06 | 0.5 | 0.67 | 0.11 | 0 | 0 | 0 | 0 | 0 | 0 | 0 | 0 | 0 | 0 |
| 14AF26 | 0 | 0 | 0 | 0 | 0 | 0 | 0.11 | 0.08 | 0.07 | 0.18 | 0 | 0 | 0 | 0 | 0 | 0 | 0 | 0 | 0 | 0 |
| 14AF27 | 0 | 0 | 0 | 0 | 0 | 0 | 0.11 | 0.18 | 0.15 | 0.17 | 0 | 0 | 0 | 0 | 0 | 0 | 0 | 0 | 0 | 0 |
| 14AF28 | 0 | 0 | 0 | 0 | 0.06 | 0 | 0 | 0 | 0 | 0 | 0 | 0 | 0 | 0 | 0.45 | 0 | 0 | 0.36 | 0.07 | 0 |
| 14AF29 | 0 | 0 | 0 | 0 | 0.08 | 0 | 0 | 0 | 0 | 0 | 0 | 0 | 0 | 0 | 0.43 | 0 | 0 | 0.29 | 0 | 0 |
| 14AF3 | 0 | 0 | 0 | 0 | 0 | 0 | 0.06 | 0.12 | 0.11 | 0.67 | 0 | 0 | 0 | 0 | 0 | 0 | 0 | 0 | 0 | 0 |
| 14AF4 | 0 | 0 | 0 | 0 | 0 | 0 | 0 | 0 | 0 | 0 | 0.09 | 0 | 0 | 0.83 | 0 | 0 | 0 | 0 | 0 | 0 |
| 14AF5 | 0 | 0 | 0 | 0 | 0 | 0 | 0 | 0.06 | 0 | 0 | 0.06 | 0 | 0 | 0.27 | 0 | 0 | 0 | 0.13 | 0.46 | 0 |
| 14AF6 | 0.1 | 0 | 0 | 0.18 | 0 | 0 | 0 | 0 | 0 | 0 | 0.4 | 0.3 | 0.6 | 0 | 0 | 0.2 | 0 | 0 | 0 | 0 |
| 14AM1 |  | 0 | 0 | 0 | 0 | 0 | 0 | 0 | 0 | 0.08 | 0.43 | 0.13 | 0.08 | 0 | 0 | 0 | 0 | 0 | 0 | 0 |
| 14AM10 |  |  | 0.1 | 0.08 | 0 | 0 | 0.4 | 0 | 0 | 0 | 0 | 0 | 0 | 0 | 0 | 0 | 0 | 0 | 0 | 0 |
| 14AM11 |  |  |  | 0 | 0.08 | 0.22 | 0.22 | 0 | 0 | 0 | 0 | 0 | 0 | 0 | 0 | 0 | 0 | 0 | 0 | 0 |
| 14AM14 |  |  |  |  | 0 | 0 | 0.08 | 0 | 0 | 0 | 0.18 | 0.57 | 0.15 | 0 | 0 | 0.5 | 0 | 0 | 0 | 0 |
| 14AM5 |  |  |  |  |  | 0.45 | 0 | 0 | 0 | 0 | 0 | 0 | 0 | 0 | 0.07 | 0 | 0.13 | 0 | 0 | 0.33 |
| 14AM6 |  |  |  |  |  |  | 0 | 0 | 0 | 0 | 0 | 0 | 0 | 0 | 0 | 0 | 0.07 | 0 | 0 | 0.08 |
| 14AM9 |  |  |  |  |  |  |  | 0.07 | 0.06 | 0.07 | 0 | 0 | 0 | 0 | 0 | 0 | 0 | 0 | 0 | 0 |
| 14JF10 |  |  |  |  |  |  |  |  | 0.67 | 0.12 | 0 | 0 | 0 | 0 | 0 | 0 | 0 | 0 | 0.08 | 0 |
| 14JF11 |  |  |  |  |  |  |  |  |  | 0.11 | 0 | 0 | 0 | 0 | 0 | 0 | 0 | 0 | 0 | 0 |
| 14JF3 |  |  |  |  |  |  |  |  |  |  | 0.06 | 0 | 0 | 0 | 0 | 0 | 0 | 0 | 0 | 0 |
| 14JF4 |  |  |  |  |  |  |  |  |  |  |  | 0.33 | 0.25 | 0.08 | 0 | 0.1 | 0 | 0 | 0 | 0 |
| 14JF6 |  |  |  |  |  |  |  |  |  |  |  |  | 0.25 | 0 | 0 | 0.5 | 0 | 0 | 0 | 0 |
| 14JF8 |  |  |  |  |  |  |  |  |  |  |  |  |  | 0 | 0 | 0.17 | 0 | 0 | 0 | 0 |
| 14JM2 |  |  |  |  |  |  |  |  |  |  |  |  |  |  | 0 | 0 | 0 | 0 | 0 | 0 |
| 14JM3 |  |  |  |  |  |  |  |  |  |  |  |  |  |  |  | 0 | 0 | 0.33 | 0 | 0 |
| 15JF2 |  |  |  |  |  |  |  |  |  |  |  |  |  |  |  |  | 0 | 0 | 0 | 0 |
| 15JF4 |  |  |  |  |  |  |  |  |  |  |  |  |  |  |  |  |  | 0 | 0 | 0.55 |
| 15JM3 |  |  |  |  |  |  |  |  |  |  |  |  |  |  |  |  |  |  | 0.1 | 0 |
| 15JM6 |  |  |  |  |  |  |  |  |  |  |  |  |  |  |  |  |  |  |  | 0 |
| 15JM8 |  |  |  |  |  |  |  |  |  |  |  |  |  |  |  |  |  |  |  |  |
